# Factors associated with HPV vaccination uptake in Uganda: a multi-level analysis

**DOI:** 10.1186/s12905-020-01014-5

**Published:** 2020-07-13

**Authors:** Alone Isabirye, Martin Mbonye, John Bosco Asiimwe, Betty Kwagala

**Affiliations:** Department of Population Studies, School of Statistics and Planning, College of Business and Management Sciences, Makerere University, Kampala, Uganda

**Keywords:** Human papilloma virus, HPV, Vaccine, Multilevel analysis, Uganda

## Abstract

**Background:**

The cervical cancer burden in Uganda is high amidst low uptake of HPV vaccination. Identification of individual and community factors associated with HPV vaccination are imperative for directed interventions. Conversely, in most Low and Middle Income Countries (LMICs) including Uganda this problem has not been sufficiently studied as the influence of individual and contextual determinants remains undetermined in spite of their substantial effect on HPV vaccine uptake. The aim of the study was to identify individual (school attendance status, age of girls, ethnicity, and amount of media exposure) and community (socioeconomic disadvantages) factors associated with HPV vaccination.

**Methods:**

Based on a modified conceptual framework for health care utilization, hierarchical modelling was used to study 6093 girls, aged 10–14 years (level 1), nested within 686 communities (level 2) in Uganda by analyzing data from the 2016 Uganda Demographic and Health Survey.

**Results:**

Majority (78%) of the girls had not been vaccinated. A number of both individual and community factors were significantly associated with HPV vaccination. The Odds of HPV vaccination were higher among girls age; 11, 13, and 14 compared to girls age 10 years, attending school compared to girls not attending school, who were; foreigners, Iteso, Karamajong, Banyoro, Basoga, and other tribe compared to Baganda, living in families with 1–8 members compared to those living in families with 9 or more members and middle social economic status compared to poor wealth quintile.

**Conclusions:**

Both individual and community factors show a noticeable effect on HPV vaccination. If higher vaccination rates are to be achieved in Uganda, these factors should be addressed. Strategies aimed at reaching younger girls, street children, out of school girls, and girls with lower SES should be embraced in order to achieve high vaccination uptake.

## Background

Worldwide, cervical cancer is the fourth most common type of cancer with 528,000 new cases annually, after lung cancer (583,100 cases), colorectal cancer (614304), and breast cancer (1,676,633 cases) [1]. Cervical cancer is responsible for 266,000 deaths among women worldwide [1]. However, the disease disproportionately affects women in limited-resource countries; almost 70% of the global burden occurs in areas with low or medium levels of human development [1]. Globally, cervical cancer is the most common cancer among women in 39 of the 184 countries and is the principal cause of cancer mortality among women in 45 countries, including Uganda. These are mainly developing countries [2]. The 2011–2020 Global Vaccine Action Plan declared a decade of vaccines vision where member states were challenged to ensure 90 and 80% national and district HPV vaccine coverage respectively by 2020 [3]. The 2013 World Cancer Declaration encouraged member states to ensure universal vaccination against HPV [4]. Additionally, goal three of the Sustainable Development Goals (SDGs) calls upon member states to reduce premature mortality from non-communicable diseases by one-third through prevention and treatment [5].

Sub-Saharan Africa has the third highest incidence (17.5%) of cervical cancer cases after India (17.7%) and East and Central Asia (18.2%). The region shares the second largest number of global cervical cancer deaths (21.6%) after India (25.4%). It is the only region where cervical cancer is equivalent to breast cancer with each constituting a quarter of the global cancer burden [2, 6]. In Sub-Saharan Africa, the East African region registers the highest number of new cervical cancer cases (52613) [7].

Uganda is among the five countries with the highest cervical cancer incidence rates in the world. It is the most commonly diagnosed cancer and has the highest incidence of malignancy and mortality among women [8]. The country’s age-standardized incidence rate of 47.5 per 100,000 is more than three times the global estimate and the country’s age-standardized mortality rate of 25 per 100,000 is more than four folds the global estimate of 6.8 per 100,000 [9]. Estimates in Uganda show that approximately 3500 women are newly diagnosed and 2400 die from cervical cancer each year. Eight out of every 10 women at the Uganda Cancer Institute are suffering from cervical cancer. Projections show that by 2025, about 6400 new cervical cancer cases and 4300 deaths will occur annually in Uganda [9].

Majority of the cervical cancer cases are potentially preventable. World Health Organization (WHO) and Uganda’s Ministry of Health (MOH) recognize primary prevention of cervical cancer i.e. preventing the initial onset of cervical cancer by vaccinating girls aged 9–14 years before exposure to sex/ HPV as a very important factor in the prevention of cervical cancer [10–12]. In 2006, the United States Food and Drug Administration (FDA) approved Gardasil; a vaccine that prevents infection with the two high-risk strains of Human Papilloma Virus (HPV) (HPV 16 and 18) recognized to cause around 70% of cervical cancers [11]. Studies have proved the cost-effectiveness of the HPV vaccine [13–15].

In Uganda, HPV vaccines against HPV 16 and 18 have been available since 2006 [16]. The first HPV pilot vaccination in Uganda was first implemented in 2008 in Nakasongola and Ibanda districts to assess the feasibility of the intervention. It was later piloted in 12 other districts in 2012 [17]. The breakthrough of these pilot projects paved the way for a countrywide rollout of the HPV vaccination in November 2015 [18]. The Ministry of Health through its strategic plan for cervical cancer prevention committed itself to achieving 80% HPV vaccine coverage among eligible girls [12]. Existing cross sectional evidence for Lira district has established low coverage (17.4%) of HPV vaccination [19] pointing to the urgent need to establish the predictors of HPV vaccine uptake.

A number of studies have examined the predictors of HPV vaccination [19–23]. Most of these studies are mainly from developed economies. Schooling status [19, 22], being older [20, 21, 23], ethnicity [20, 24, 25], medium social economic status [21, 23, 25] were significantly associated with HPV vaccination. These studies focused on the associations between individual-level factors and HPV vaccination with an assumption of independence of errors which is partly realistic. They did not segregate the effect of individual and community factors on HPV vaccination even when they dealt with data of hierarchical nature. Most of those previous studies overlooked the significance of contextual phenomena since community-level determinants were not appropriately considered in their analyses. It is important to put contextual phenomena into consideration as people dwelling in the same neighborhood tend to exhibit similarities with respect to their health outcomes. For that reason, it isessential to consider contextual factors either at the design and/or analytical phase to understanding individual health outcomes in a population. In Low and Middle Income Countries (LMICs), HPV vaccination is yet to be sufficiently examined by multilevel analysis, an analytic approach that takes care of both random and fixed effects in a single model. Multilevel analysis facilitated us to detach the effect of individual and community factors on HPV vaccination based on the level at which they shaped HPV vaccination. In contrast, the deployment of single-level analyses (individual or ecological analyses) instead of multilevel analyses presents challenges in inferring whether community-level determinants affect HPV vaccination uptake notwithstanding the individual factors or whether inter-community variation in HPV vaccination is entirely influenced by individual characteristics without any influence of community-level determinants. Additionally, there is growing evidence of associations between community-level factors and HPV vaccination after considering individual factors [26]. The present study seeks to investigate whether HPV vaccination can be predicted by personal and community determinants using a multi-level model.

## Methods

The study used secondary data from the 2016 Uganda Demographic and Health Survey (UDHS). Permission to access the UDHS data was sought from Measure DHS [27]. The UDHS employed a cross-sectional survey that applied a stratified two-stage cluster sampling design [28], which was used in the 2014 population and housing census [29]. A comprehensive explanation of sampling approach is published in the UDHS report [28]. The 2016 UDHS household members’ recode contains data of 91,167 household members age 0–98 years. We selected girls age 10–14 years who were eligible for HPV vaccination module and the household respondent answered the question “has (name) ever had HPV Vaccine to prevent cancer?”. This resulted into a weighted sample of 6093 girls [27].

### Measure of outcome variable

The outcome variable “HPV vaccination” was measured using the question: “has (name) ever had HPV Vaccine to prevent cancer?” (No/Yes). This question was asked eligible household respondents who were parents or guardians of girls age 10 to 14 years.

### Explanatory variables

**Fig. 1:**
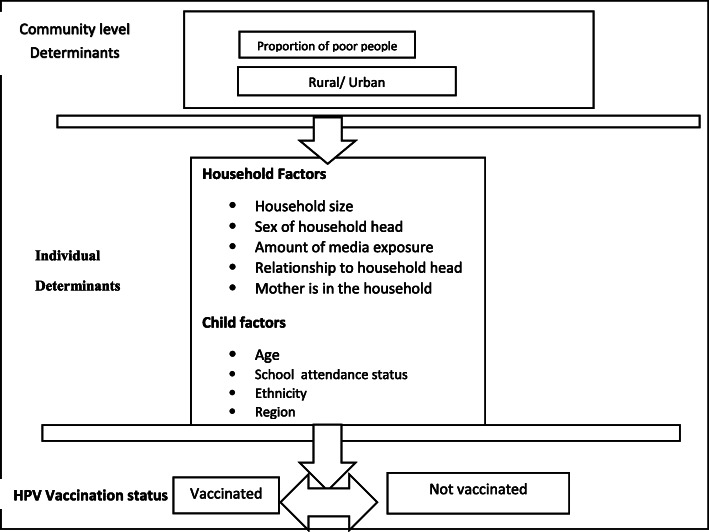
Conceptual Framework for individual and community-level determinants influencing HPV vaccination

Individual and community characteristics that were examined for possible associations with HPV vaccination were based on a framework with components adapted from Anderson -Newman behavioral model of health services utilization and Bandura’s social cognitive theory [30, 31]. This framework was developed taking into account the available information in the 2016 Uganda Demographic and Health Survey. The adapted framework for HPV vaccination is depicted in Fig. 1.

### Individual-level determinants

Individual-level variables included girls’ age [10–14], currently attending school (Yes/ No), ethnicity (Baganda, Foreigners, Luo, Lugbara, Iteso, Karamajong, Banyankole, Banyoro, Basoga, and Others), region (Western, Central, Eastern, Karamoja, and Northern), sex of household head (Female/ Male), number of members in the household (1–8 and ≥ 9 members), relationship to the household head (daughter and other relationship) and living with mother in the household (No/ Yes). Access to media was assessed using amount of media exposure. Amount of media exposure was obtained using data on a households’ ownership of media types such as televisions, radios, and telephones. For this study, amount of media exposure was categorized into 0, 1, and ≥ 2 types of media.

### Community level determinants

The community (cluster) was used as the primary sampling unit (PSU) of the data. The community influence on HPV vaccination was measured by considering the socioeconomic status of the community in which the girls were living. The community socio-economic disadvantage was operationalized by combining two factors: place of residence (rural/urban) and wealth index (poorest, poorer, middle, and rich quintile). These variables were obtained by combining individual answers for each question to the cluster (community) level. The Principal Component Analysis (PCA) was used to generate community wealth quintiles. A number of studies have applied community wealth quintile as a community-level determinant [32, 33].

### Statistical analyses

We used frequency distributions to describe the demographic and socioeconomic characteristics of the girls. Associations of individual and community level characteristics with HPV vaccination (predicted variable) were investigated using cross-tabulations. We used Pearson’s chi-squared (*x*^2^) tests to examine the independent predictors of HPV Vaccination and the level of statistical significance was set at *p* <  0.05. Data analysis was guided by the framework for health care utilization and the hierarchical nature of the Uganda DHS data. Thus, we used the three-step multi-variable multilevel logistic regression with the log-binomial function of the generalized linear mixed models family [34]. The associations of individual-level and community-level determinants with HPV vaccination were analyzed in a stepwise manner. The nesting of individual-level determinants within community-level determinants in which girls live generated three models for analysis. We started by fitting the variance component model or empty model (null model); the empty model excluded the fixed effects. The variance component model was constructed to determine whether the variation in HPV vaccination could be explained by variations in communities in which girls live (model including random effects only). This was attained by establishing the Intra-Cluster Correlation coefficients (ICCs)/ Variance Partition Coefficients. The ICCs are obtained by dividing the proportion of variance at the group level with the total variances at the individual and group levels [35]. We fitted model 2 adding all the individual-level factors. Finally, model 3 was fitted comprising of individual-level and community-level determinants. To assess the fitness of model 3 relative to model 2, we estimated the likelihood ratio test and Akaike Information Criterion (AIC) of the two models; with a lower AIC value denoting a better model fit [36]. The odds of HPV vaccination while controlling for individual-level and community-level determinants in model 3 were presented with their accompanying *P*-values and 95% confidence intervals [37]. We performed Variance Inflation Factor (VIF) and Tolerance test to check for multicollinearity among the covariates in the models. No multicollinearity problems were observed in the regression models since all variance inflation factor values were less than 10 and tolerance values were greater than 0.1. Stata SE 15 software was deployed for the analyses and the two-tailed Wald test was used to determine the statistical significance of the covariates at significance level of alpha equal to 5% [35].

### Ethical considerations

All data that was used in the study were obtained from the 2016 UDHS. During data collection, written informed consent was obtained from each respondent before the interviews [28]. We obtained approval to use the data from the DHS repository (http://dhsprogram.com/data/available-datasets.cfm).

## Results

### Descriptive characteristics

The general characteristics of the study population are shown in Table 1. About 74% of the girls were below 13 years, 1 in three (30.5%) were from the Eastern region and a larger proportion (82.9%) were from rural areas. Approximately a quarter (24.9%) of the girls were in the wealth quintile of poorest. Most of the girls (89.9%) were attending school. The majority were living in male headed households (66.6%) with 5–8 household members (59.5%). The majority lived in households with access to media (90.9%). Majority (69.6%) were daughters to household heads and were living with their mothers (66.6%). More than two thirds (78%) (results not shown in Table 1) had not received the HPV vaccine.

**Table 1:** Distribution of girls by their demographics, socioeconomic factors and HPV vaccination (*N* = 6093)

Characteristic	% OF GIRLS	Frequency	(%)Vaccinated	*P*-value
**Age**				
10	24.7	1504	19.5	0.034
11	16.8	1023	23.4	
12	21.7	1322	21.1	
13	20.9	1271	23.8	
14	16.0	973	23.3	
**Region**				0.010
Kampala	3.3	203	18.2	
Central	14.0	854	18.2	
East	30.5	1859	22.6	
Karamoja	5.7	349	28.9	
North	21.9	1332	22.4	
West	24.6	1496	20.3	
**Type of residence**				0.482
Urban	17.7	1040	21.1	
Rural	82.9	5053	22.1	
**Currently attending school**				< 0.0001
No	10.1	617	15.1	
Yes	89.9	1246	22.7	
**Sex of household head**				0.117
Male	66.6	4057	21.5	
Female	33.4	2036	23.0	
**Has disability**				0.246
No	97.1	5914	22.1	
Yes	2.9	179	18.4	
**Relationship to household head**				0.016
Daughter	69.6	4242	22.8	
Other relationship	30.4	1851	20.0	
**Amount of media exposure**				0.447
0	19.2	1168	21.4	
1	51.4	1961	21.3	
≥ 2	29.4	2964	22.6	
**Mother is in the household**				0.017
No	33.4	2036	20.2	
Yes	66.6	4057	22.9	
**Number of household members**				0.028
1–8	74.4	4532	22.7	
9+	25.6	1561	20.0	
**Wealth index**				0.243
Poorest	24.9	1516	21.1	
Poorer	20.4	1243	20.8	
Middle	21.2	1290	23.7	
Rich	33.4	2044	22.3	
**Ethnicity**				< 0.0001
Foreigners	0.9	55	34.6	
Baganda	13.4	814	19.7	
Luo	18.2	1110	21.9	
Lugbara	4.6	281	21.4	
Ateso	9.0	550	23.8	
Karamajong	4.5	275	30.6	
Banyankole	15.1	917	16.7	
Banyoro	5.7	350	24.9	
Basoga	6.3	384	27.3	
Other	22.3	1357	21.9	

### Association of Individual-level and Community level characteristics with HPV vaccination

Table 1 shows the findings of the cross tabulation (Chi-square tests) of individual and community explanatory variables with HPV vaccination. HPV vaccination was significantly associated with the age of girls, region, schooling status, relationship to household head, number of household members, and ethnicity. HPV vaccination was higher among girls age 13 years (23.8%), in Karamoja region (23.8%), girls who were attending school (22.7%), and those who were living with their mothers in the household (22.9%). It was also relatively high among girls who were daughters to the household head (22.8%). Vaccination was high among girls who lived in households with 2 or more types of media (22.6%) and with less than 8 members (22.7%). HPV vaccination was relatively high among foreigners (34.6%). Type of place of residence, sex of household head, disability status, amount of media exposure, and wealth index were not significantly associated with HPV vaccination.

The results of multi-level analysis are presented in Table 2. The null model (empty model) which is also referred to as variance component model (results not shown in Table 2) was used to determine the total variance in HPV vaccination that is due to the communities in which the girls were living. There was significant (*P*-value < 0.001) variation in HPV vaccination at community-level. The study findings show that community-level determinants partly account for the total variance in HPV vaccination hence the community-level determinants were sufficiently catered for by the Multivariable Multilevel Regression Analysis (MMLRA). Our variance partition coefficient (VPC) or intra-cluster correlation (ICC) of 0.56 indicate that the communities in which the girls live contribute to 56% of the variation in HPV vaccination. This also suggests that the intra-community correlation amongst girls vis-à-vis the likelihood of HPV vaccination was 0.56.

**Table 2:** Associations between individual and community factors with HPV Vaccination

	Model 2 including individual level determinants		Model 3 including individual and community level determinants	
Fixed effect (OR, 95% CI)				
**Individual-level determinants**				
**Girl’s age**				
10 (Ref)				
11	1.31	(1.06–1.61)*	1.30	(1.06–1.61)*
12	1.15	(0.94–1.40)	1.14	(0.94–1.39)
13	1.41	(1.15–1.71)**	1.40	(1.15–1.70)**
14	1.42	(1.15–1.76)**	1.41	(1.14–1.75)**
**School attendance status**				
No (Ref)				
Yes	2.93	(2.16–4.0)^***^	2.88	(2.12–3.92)***
**Ethnicity**				
Baganda (Ref)a				
Foreigners	3.31	(1.71–6.42)***	3.33	(1.72–6.45)***
Luo	1.15	(0.71–1.86)	1.18	(0.73–1.91)
Lugbara	1.17	(0.65–2.11)	1.21	(0.67–2.18)
Iteso	1.73	(1.13–2.64)^*^	1.79	(1.17–2.73)^**^
Karamajong	3.66	(1.49–8.97)**	3.84	(1.57–9.43)^**^
Banyoro	1.52	(1.05–2.21)*	1.54	(1.06–2.23)^*^
Basoga	2.18	(1.43–3.32)***	2.15	(1.41–3.28)***
Other	1.48	(1.11–1.98)^**^	1.50	(1.12–2.01)^**^
**Region**				
Western (Ref)				
Central	0.97	(0.73–1.29)	1.00	(0.74–1.33)
Eastern	0.81	(0.58–1.12)	0.82	(0.59–1.14)
Karamoja	1.29	(0.56–2.95)	1.36	(0.59–3.14)
Northern	1.24	(0.77–2.00)	1.31	(0.81–2.12)
**Household characteristics**				
Amount of media exposure				
0 (Ref)				
1	1.10	(0.90–1.35)	1.03	(0.83–1.28)
≥ 2	1.22	(1.00–1.50)	1.12	(0.87–1.41)
**Sex of household head**				
Female (Ref)				
Male	0.87	(0.75–1.01)	0.87	(0.75–1.01)
**Number of members in the household**				
1–8 (Ref)				
≥ 9	0.81	(0.69–0.96)*	0.81	(0.69–0.95)**
**Relationship to household head**				
Daughter (Ref)				
Other relationship	0.94	(0.75–1.18)	0.94	(0.75–1.18)
**Mother in the household**				
No (Ref)				
Yes	1.16	(0.94–1.44)	1.17	(0.94–1.46)
**Community level factors**				
**Type of residence**				
Rural (Ref)				
Urban			0.92	(0.72–1.17)
**Wealth index**				
Poorest (Ref)				
Poorer			1.11	(0.88–1.40)
Middle			1.31	(1.01–1.69)*
Rich			1.22	(0.93–1.59)

*p < 0.05, **p < 0.01, ***p < 0.001

Ref = Reference Category

OR = Odds Ratios

CI = Confidence Interval

The estimated community variance was also presented as median odds ratios (MOR = 0.24) which means that girls from an average community in Uganda had 24% less odds of having already been vaccinated. After the decomposition of HPV vaccination in model 1, level one fixed effects (individual-level covariates) were added into the empty model to form model 2. The community-level variance increased in model 2 which means that the frequency of individual factors is different in all communities in Uganda. After considering both individual and community-level characteristics in model 3, it was observed that the community-level variance reduced marginally in model 3. This showed that the frequency of community factors is almost similar in all communities in Uganda. After the addition of both individual and community-level factors, the variation in HPV vaccination behavior among communities remained significant. The estimated ICC show that the variability (54%) in HPV vaccination was due to community differences (ICC = 0.54, *P* <  0.0001). It is worthy to state that a random intercept model was considered rather than the usual single-level model due to the hierarchical nature of the data and to avoid biased associations.

### Fixed effects (measures of associations)

Table 2 presents the fixed effects for individual and community-level factors. The fixed effects presented in model 2 show the associations between HPV vaccination and individual-level factors prior to consideration of community-level covariates. The fixed effects presented in model 3 indicate the associations between HPV vaccination and both individual and community-level factors. Subsequent consideration of both individual and community-level characteristics in model 3 indicated that a number of fixed effects (age, school attendance, being; a Foreigner, Iteso, Munyoro, Karamajong, Musoga and other tribe) steadily maintained their significance after adding level two fixed effects (community level factors). The variables for being an Iteso and having 9 or more members living in a household also remained statistically significant after controlling for level two fixed effects. The analysis of only individual-level factors, showed that child’s age, school attendance, ethnicity, and size of family were significantly associated with HPV vaccination; the intra-class correlation coefficient (ICC) showed that 54% of the variance in HPV vaccination was due to common community characteristics (ICC = 0.54, *p* <  0.0001).

In the final model (Table 2), we included both individual- and community level characteristics. The results show that odds of HPV vaccination were higher among girls attending school (OR = 2.88; 95% CI 2.12–3.92) than those who were not attending school. In respect to age, odds of HPV vaccination were higher among girls age 11, 13, and 14 years with OR = 1.30; 95% CI 1.06–1.61, OR = 1.40; 95% CI 1.15–1.70, and OR = 1.41; 95% CI 1.14–1.75 respectively compared to girls aged 10 years. In relation to tribe, odds of HPV vaccination were higher among girls who were foreigners, Iteso, karamajong, Banyoro, Basoga, and other tribe with OR = 3.33; 95% CI 1.72–6.45, OR = 1.79; 95% CI 1.17–2.73, OR = 3.84; 95% CI 1.57–9.43, OR = 1.54; 95% CI 1.06–2.23, OR = 2.15; 95% CI 1.41–3.28, and OR = 1.50; 95% CI 1.12–2.01 respectively compared to Baganda girls. Odds of HPV vaccination were lower among girls who were living in households with 9 or more members (OR = 0.81; 95% CI 0.69–0.95) compared to those who were living in households with 1 to 8 members. Odds of HPV vaccination were higher among girls who were living in communities with middle wealth quintile (OR=; 95% CI 1.01–1.69) compared to those who were living in communities with the poorest wealth quintile.

## Discussion

According to the study findings, uptake of the HPV vaccine among Ugandan girls aged 10 to 14 years was low (22%). Although MOH had committed itself to achieve 80% HPV vaccine coverage by 2015 [12], one year before the survey [28]. These findings are close to the findings of a cross sectional study from northern Uganda [19]. Uganda’s HPV vaccine coverage is lower than that of Rwanda (93.2%) [38]. This low HPV vaccine coverage in Uganda could be associated with negative attitudes towards the vaccine [19, 39], limitations associated with the school based HPV vaccine delivery strategy [13, 17, 40, 41], and social cultural factors [41].

This study established the impact of contextual factors besides individual characteristics on HPV vaccination. Our findings established that the likelihood of HPV vaccination was not solely shaped by individual characteristics, but also communities where these girls were residing. Both community and individual-level factors were significantly associated with HPV vaccination. The study results found a significant negative association of socioeconomic deprivation of communities (rural areas with high proportion of poor people) with HPV vaccination. The strength of deprivation is determined by those two elements of socioeconomic disadvantages though they don’t coexist together in similar proportions. There is scanty evidence in LMICs regarding the relationship between community level characteristics and HPV vaccination yet findings indicate that community level characteristics strongly predict health care utilization [21, 30–33]. The plausible explanation for this association is that people dwelling in the same community with socioeconomic disadvantages always have similar health care utilization (HPV vaccination) behaviors. People sharing community socioeconomic disadvantages tend to have challenges in accessing health care services, education and appreciating the significance of health care services. Community factors will mediate through individual level factors to influence health care utilization (HPV vaccination).

Our study indicate that older girls were more likely to be vaccinated than their one year younger counter parts. These findings are in consonance with earlier studies [21, 23, 42]. However, our findings are not supported by studies conducted elsewhere; both the oldest and the youngest age categories were found to have lower likelihood of HPV vaccination in the Netherlands [43]. This age effect may be attributed to an increased acceptance of the vaccine by the parents among their older daughters [44]. Another probable reason is procrastination: With HPV vaccine, girls have a long time lag (9–14 years) of eligibility for vaccination [12]. Girls might be reluctant to vaccinate at the lowest eligibility age. Finally, the sensitization posters or messages by which girls were informed about their eligibility (9–14 years) for HPV vaccine may have had a procrastination effect on vaccination initiation. This is consistent with previous research in which patient reminder and recall systems have been established to affect vaccination behavior [45].

The results of this study indicate that School attendance status was positively associated with HPV status. These findings are consistent with prior studies [19, 22]. The plausible explanation for this association is the implementation of the school based HPV vaccine delivery strategy without special effort to reach out of school girls [20, 22, 25].

The current study found that ethnicity was significantly associated with HPV vaccination. This finding is similar to previous studies [21, 23, 25, 43]. The probable reason for this association is that individuals belonging to a social group with low uptake of vaccination have a higher chance to come across damaging beliefs, norms and emulate behavior from their peers. Another important finding was that medium social economic status was positively associated with HPV vaccination. The likelihood of having already been vaccinated was found among girls from middle wealth quintile settings although vaccination was free of charge for all girls. Such association between socio-economic status and adolescent vaccination has been found in other studies [23, 25, 43, 46]. The probable explanation for this association is that HPV vaccine was rolled out nationwide in 2015 [18] one year before the survey [28] making it relatively new. Adoption of new positive health behaviors has been associated with Social Economic Status (SES) [47]. People with low SES are likely to adopt a new positive health behavior last because they base their decisions on what happened in the past, change behavior a long time after changes in their awareness and knowledge, suspicious of new interventions, take more time to convince and often poor economic position makes them very cautious [47]. However, the current findings are not consistent with some previous findings. Socioeconomic status was found not to be significantly associated with HPV Vaccination [22].

### Study limitations

Notwithstanding the strength of this study, there were some limitations with the data. Our study was based on cross-sectional and secondary data. The dataset had no variables on mother’s characteristics to facilitate better assessment of mother’s characteristics. The dataset had no information for 9 year old girls to facilitate better assessment of vaccination coverage among girls age 9 years. Second, we combined individual responses to generate our measures at community level. It is therefore difficult to ascertain whether some girls were not classified into wrong administratively demarcated boundaries (clusters). The use of hierarchical regression models require aggregating individual responses to community level assuming that the groups are homogenous. This has potential consequences on the interpretation of results because associations at aggregated levels may not directly apply to individuals but to the group of individuals with in a given area. Nevertheless, the current study points to important programmatic areas of intervention for promoting HPV vaccination in Uganda.

## Conclusion

This study considered countrywide representative data on HPV vaccination for the 2016 Uganda demographic and health survey. The results of the study established low HPV vaccine coverage in Uganda. Both community (community socioeconomic disadvantage) and individual (school attendance status, age of girls, ethnicity, and amount of media exposure) level factors were found to be significantly associated with HPV vaccination. Other countries in the region with organized school-based programmes have had much higher uptake rates. If higher vaccination rates are to be achieved in Uganda, both individual and community level factors responsible for variation in HPV vaccination should be addressed. System-wide interventions should be implemented to increase vaccine coverage in Uganda. Our findings point to the need for universal basic education, creation of job opportunities, and poverty alleviation. Our findings further suggest that effort should be directed at women and rural affirmative interventions to narrow gender and type of residence inequality gaps respectively. These are vital interventions that can be implemented at community level to mitigate the effects of community socioeconomic disadvantages. Variation in HPV vaccination among different ethnic groups indicate that communication on HPV vaccine should be tailored to ethnic communities.

## Data Availability

Data are from the Demographic and Health Survey. The dataset is open to qualified researchers free of charge. To request access to the dataset, please apply at http://dhsprogram.com/data/Access-Instructions.cfm.

## Abbreviations

CI: Confidence interval
FDA: Food and Drug Administration
HPV: Human papillomavirus
MOH: Ministry of Health
OR: Odds ratio
SDGs: Sustainable Development Goals
SES: Socio-economic status
UBOS: Uganda Bureau of Statistics
